# Process analysis of solar steam reforming of methane for producing low-carbon hydrogen

**DOI:** 10.1039/c9ra09835f

**Published:** 2020-03-27

**Authors:** Enkhbayar Shagdar, Bachirou Guene Lougou, Yong Shuai, Enkhjin Ganbold, Ogugua Paul Chinonso, Heping Tan

**Affiliations:** Key Laboratory of Aerospace Thermophysics of Ministry of Industry and Information Technology, Harbin Institute of Technology 92 West Dazhi Street Harbin 15001 China shuaiyong@hit.edu.cn bachguelou@yahoo.fr; School of Energy Science and Engineering, Harbin Institute of Technology (HIT) 92 West Dazhi Street Harbin 15001 China; School of Power Engineering, Mongolian University of Science and Technology (MUST) Ulaanbaatar 14191 Mongolia; School of Mechanical and Power Engineering, Harbin University of Science and Technology (HUST) Nangang District Harbin 15001 China

## Abstract

Regarding the trend of hydrogen-powered fuel cell engine development, hydrogen fuel is undisputedly the next generation renewable and sustainable energy carrier. The steam reforming of methane (SRM) is a field-proven technology for efficient hydrogen production. However, producing low-carbon hydrogen is the most technical challenge related to available hydrogen production technologies. This paper investigated the process analysis of SRM for low-carbon hydrogen production using concentrated solar energy as a heat source. Analysis of the solar SRM is carried out considering the reformate gas and their influencing factors. The operating temperature of 200–1000 °C and the pressure of 1.02–30 bar were considered when the mass ratio of steam-to-methane in feed gas was varied from 1.0 to 4.0. It was found that the composition of reformate gas, hydrogen yield, methane and steam conversion rate, the thermal efficiency of reforming reactor, and volume flow of reformate gas are significantly affected by the operating parameters including temperature, pressure, and the mass ratio of feed gas. Carbon content in the yield of hydrogen produced can be limited by considering the water–gas shift reaction in the SRM process. Besides, the centralized tower type solar concentrating system is selected as the heat source of the SRM process. The effect of solar radiation on the operation performance of the solar SRM process was analyzed. Direct normal irradiation is a key factor affecting the operating performance of the solar SRM process.

## Abstract 2

Integrating solar thermal energy into conventional SRM technology is a promising approach for low-carbon hydrogen production based on fossil fuel in near and midterm.

## Graphical abstract

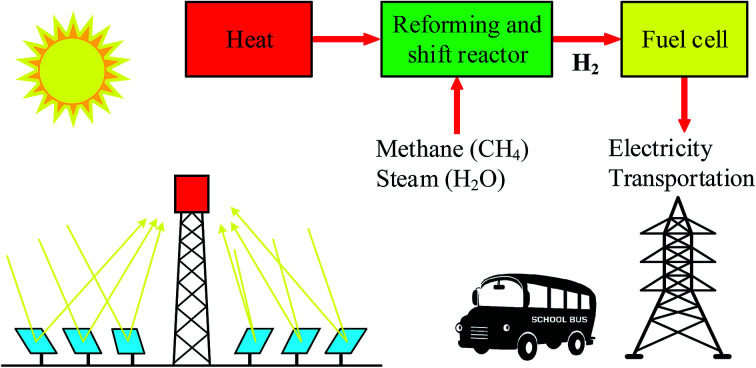

## 1. Introduction

Developing clean fuel production technology is a promising way to meet the worldwide increasing energy demands with a great contribution to mitigating the raising effect of greenhouse gases and environmental pollution hazards. Apart from increasing environmental pollution effects, the prices of fossil fuels such as natural gas and oil are increasing unceasingly while its reserves are decreasing. According to the statistical review of world energy 2018, the reserves of natural gas (NG) and oil are limited to a range of 40–60 years while the reserves of coal can last for more than 150 years.^1^ The increasing interest in the issues related to environmental pollution and economic growth have recently led to the development of next-generation sustainable and renewable clean fossil fuel production as energy carriers. Regarding a range of products that are compatible with our energy infrastructure today, hydrogen can be considered as the next-generation energy carrier since hydrogen is one of the important feedstock materials in chemical, petroleum, metallurgical, and electronic industries. In the transportation sector, hydrogen is commonly used in a fuel cell system based on electrochemical conversion and an internal combustion engine system *via* direct combustion. The transportation sector accounts for approximately 60% of the total air pollution.^2^ For that reason, all vehicles with conventional internal combustion engine systems are needed to be replaced by the hydrogen and electric engine systems. Sufficient technologies have been developed for hydrogen production. However, steam reforming of methane process is considered as state-of-the-art technology for efficient production of hydrogen regarding the current scientific research and development (R&D). In the SRM process, pure methane and natural gas are not only used for producing hydrogen. Moreover, other types of gases with higher methane content (marsh gas, biogas, raw syngas from gasification, coal mine methane, and coal bed methane) are possible to be used for increasing the quality of syngas and producing hydrogen. Integrating solar thermal energy into conventional SRM technology is a promising approach for ecological hydrogen production based on fossil fuel in the near and midterm. In the long term, the solar-based full green and clean technologies, including thermochemical,^3^ photochemical,^4^ electrochemical^5^ and water electrolysis will be dominated for producing hydrogen.

In recent years, intensive researches have been developed on different methods and technology under various conditions for methane reforming technology development. Pure hydrogen production technology based on the reforming process consists of converting methane into a hydrogen-rich gas, which is further upgraded and purified to hydrogen. Different types of reforming processes such as steam reforming (SRM), dry reforming (DRM), partial oxidation (POX), auto-thermal reforming (ATR), combined reforming (bi and tri), and plasma reforming have been investigated for efficient hydrogen production.^6–8^ Regarding the steam reforming process, methane (natural gas) reacts with steam to produce hydrogen-rich gas using appropriate catalysts at a temperature ranging from 200–1000 °C and operating pressure ranging from 1–30 bar. The steam reforming process is always associated with the water–gas shift (WGS) reaction, which generates a high amount of CO_2_. Steam methane reforming and WGS reactions are the global reactions for leading to a significant amount of hydrogen production. Considering steam methane reforming reaction, WGS reaction can reduce the concentration of CO in the amount of gaseous product. After the WGS reaction, the concentration of CO in the reformate gas is lower than 1.0%. Water–gas shift reaction is an exothermic type reaction. However, this might lead to excessive CO_2_ generation in hydrogen-rich gas produced.^6,7^ Steam methane reforming reaction can be typically described by the following equations.

Water–gas shift reaction (CO-shift reaction):

1 CO + H_2_O ↔ CO_2_ + H_2_; Δ*H*_298_ = −41 kJ mol^−1^

Steam methane reforming reaction (SRM#1):

2 CH_4_ + H_2_O ↔ CO + 3H_2_; Δ*H*_298_ = 206 kJ mol^−1^

Overall reaction (SRM#2):

3 CH_4_ + 2H_2_O ↔ CO_2_ + 4H_2_; Δ*H*_298_ = 165 kJ mol^−1^

**Fig. 1:**
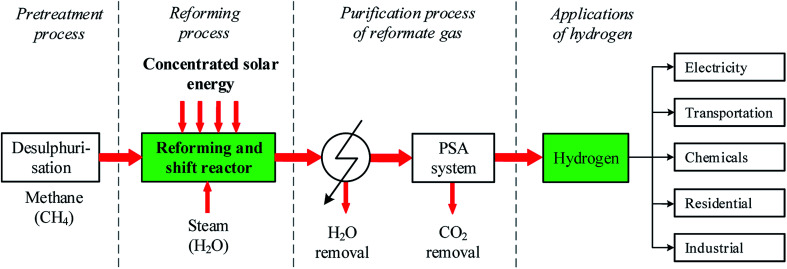
Solar SRM process and its application.

Since the past decades, the R&D on SRM technology aims to produce a high concentration hydrogen-rich gas which can be commonly used as an important feedstock in the chemical industry, energy, and transportation sector for various purposes. In the SRM process, the composition of the reformate gas, especially hydrogen yield depends on the reaction temperature and the operating pressure, process time, the quality of the feedstock, the catalyst activity, the mass ratio of steam-to-methane (H_2_O/CH_4_) in the feed gas, and the type of reactor design. Finding appropriate candidate catalysts for the SRM process is a big technical challenge since the use of a potential catalyst can significantly improve the performance of thermal chemical reactivity thereby resulting in an important amount of reformate gas production. In recent years, researchers have significantly advanced the structure of the reactor and the catalyst materials. Among common-based catalyst materials including Ni, Fe, Co, and Al, other types of catalysts including Ru, Rh, Pd, Ir, and Pt have been investigated for higher hydrogen production and greater durability.^9–11^Fig. 1 describe the solar SRM process and different application fields for H_2_ production. As indicated in Fig. 1, the steam reforming process is mainly a composite of feedstock pretreatment (desulfurization) process, reforming and shift reactor and purification process of reformate gas.

Hydrogen production *via* SRM is a high endothermic process which requires a high potential heat source for preheating feedstock, producing high potential steam, and controlling steam reforming process. However, developing medium and high-temperature scales concentrated solar energy system is another challenge that can overcome this issue by contributing to CO_2_ emission reduction. Solar steam reforming methane is one of the energy effective technologies for producing low carbon hydrogen from methane.^12^ The use of concentrated solar energy for a high-temperature heat source to the SRM process has the potential of avoiding up to 35–40% of the CO_2_ emissions derived from the conventional SRM process based on fossil fuel.^13,14^ Last decade, many studies have been developed on effective and reliable technology of the solar SRM under different methods. Wang *et al.* have performed thermodynamic analysis and numerical investigation of concentrated solar-powered membrane reactor concept for hydrogen production at lower and medium operating temperatures based on SRM.^15^ Moreover, Giaconia *et al.* have investigated the solar steam reforming of natural gas for hydrogen production using molten salt heat carriers.^16^ Besides, a dynamic simulation model is developed on a 400 kW solar reforming reactor to predict the temperature variation, heat transfer rates, outlet mass flow rates, and product composition during purging, thermal testing, experimental run, and cycling operation.^17^ A numerical model combining Monte-Carlo ray-tracing method with a finite-element method is developed for the thermodynamic and kinetic analysis of solar methane reforming reactor using a reticulated porous ceramics structure of Ni/CeO_2_–ZrO_2_ as a potential catalyst. Analysis of the system is performed under different reaction conditions with wide ranges of temperature, solar power input, and the ratio of steam to methane.^18^ Moreover, Brown *et al.*^19^ have developed the studies on the potential cost, performance, and economic characteristics of steam reforming of methane with parabolic dish concentrator for syngas production. The evaluation and performance analysis of membrane reactor with natural gas and steam for hydrogen production was investigated using concentrated solar energy as an energy source, molten salt thermal storage process, and Ni-based catalyst.^20^ The thermodynamic^21^ and economic analysis^22^ of solar steam reforming of NG integrated with a gas turbine power plant has been investigated. Regarding carbon capture and utilization technology, DRM process can significantly contribute to CO_2_ emission reduction. Performance analysis of solar thermochemical reactor was investigated based on the solar-driven methane reforming process using CO_2_.^23,24^ The solar-thermal fluid-wall aerosol flow reactor powered by high-flux concentrated solar energy was designed for the investigation of carbon black and hydrogen production based on the DRM process.^25^ Intensive studies have been conducted on the numerical investigation of the thermochemical analysis^26^ and thermochemical storage performance analysis^27^ of DRM in a volumetric foam reactor powered by concentrated solar radiation.

In this section, some examples of process analysis of simulation models for SRM available in the literature are introduced. The simulation models based on equilibrium constant, and kinetic method using some process simulation software have been performed for the thermodynamic analysis of the SRM process. The effect of different factors including temperature, pressure, and the mass ratio of steam-to-methane on the steam reforming of NG for hydrogen production was studied. Two equilibrium reactions, such as steam methane reforming reaction and WGS reaction for the conversion of steam and carbon monoxide to hydrogen and carbon dioxide were examined.^28^ The steady-state simulation model was developed for hydrogen production *via* steam reforming of NG using Aspen Hysys software. The optimization and sensitivity analysis of hydrogen production based on steam reforming process has been studied.^29^ Moreover, the process simulation software Aspen Plus was used to perform sensitivity analysis and optimization of hydrogen production based on SRM.^30^ Furthermore, Gibbs free energy minimization methods *via* Aspen Hysys was developed to numerically investigate the thermodynamic analysis and optimization of dry and partial oxidation and auto-thermal (combined DR and POX) reforming processes at temperatures range of 200–1000 °C and pressure range of 1–3 bar.^31^ The effect of several parameters including the process temperature, operating pressure, catalyst weight loading, and mass ratio of feed gas on the process of steam reforming of NG has been studied to perform hydrogen production. The kinetic-based simulation model of steam methane reforming and WGS reaction for hydrogen production was performed in Aspen Plus.^32^ The simulation model of DRM and SRM for syngas production using solar or hybrid heat sources was developed and investigated.^33^ Solar cracking and solar SRM processes have been compared with the conventional SRM based on hydrogen production material and energy source.^34^ The process analysis and simulation model of SRM, POX and ATR processes *via* the solar thermochemical hydrogen production was developed and studied by Aspen Plus software.^35^

As resulted from literature reviews, solar energy is possible to use for driving endothermic steam reforming reactions in which methane is reacted to form hydrogen-rich gas. In recent years, many studies have been conducted on the solar SRM process using different methods and technologies. The most of previous studies of solar SRM are investigated using numerical simulation of the CFD model and laboratory-scale experimental setup. However, the previous studies on the process analysis for solar SRM are not comprehensive. Therefore, it is practically important to study the process analysis of solar SRM. Moreover, the study of a large scale commercial solar SRM plant with a concentrated solar system is significantly important.

In this study, the simulation study of solar SRM for hydrogen production is investigated using Pyrolysis and Gasification Process Library and Concentrating Solar Power Library in IPSEpro process simulation software. This study aims to find the thermodynamically favorable operating conditions on the solar SRM process. Therefore, the effect of operating parameters on the SRM process is considered. The effect of several operating parameters, including temperature, pressure, and mass flow ratio of the feed gas is analyzed. Moreover, the solar SRM plant for processing 5.0 tons of methane per hour is investigated based on the estimated optimum operating conditions. The centralized tower type solar concentrating system is selected as the heat source of solar SRM. The effect of solar radiation on the operation performance of solar SRM process is considered.

## 2. Methods

### 2.1. Model of steam reforming and water–gas shift reactor

**Fig. 2:**
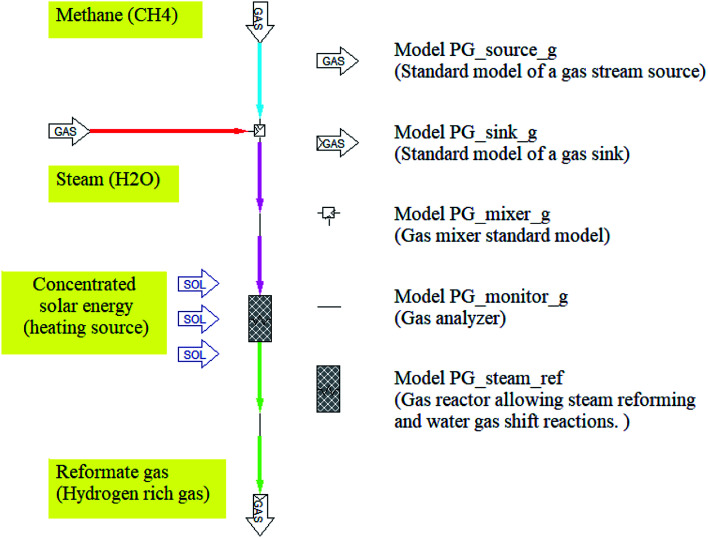
Simulation model for the steam reforming of methane.

The thermodynamic equilibrium in steam reforming methane reactor can be calculated in two methods. One is to minimize the Gibbs free energy, the other is to use equilibrium constants. According to the previous studies, the steam methane reforming and water–gas shift reactions were modeled based on the method of equilibrium constants. In this study, the simulation model of solar SRM for hydrogen production is investigated using IPSEpro process simulation software based on the method of equilibrium constants. The simulation model of SRM reactor was performed using the equilibrium type reactor, thus there is no catalyst effect on the final equilibrium composition of the reformate gas from the reactor. The simulation model of SRM is one-dimensional model, which is not considered any dimensions, reactor design, velocity, reaction rate, and catalyst effect. Pyrolysis and Gasification Process library (PGP library) of IPSEpro process simulation software^36^ is used to investigate the process analysis of steam reforming of methane for hydrogen production. The unit model of steam reformer is a model for various applications. The model equations are accessible for the steam reforming methane process. In this unit model, both the steam methane reforming and the water–gas shift reaction can take place simultaneously and allow us to calculate equilibrium as well as non-equilibrium gas compositions at the given temperature and ratio of H_2_O/CH_4_. The process flow diagram of SRM and component models used in the simulation model are basically described in Fig. 2.

CH_4_ and H_2_O considered as feeding streams were firstly mixed by a mixer standard model before entering into the chemical reactor. The inlet and outlet streams are analyzed and monitored by the gas analyzer installed at the inlet and outlet of the gas reactor. The component model of the gas-phase chemical reactor has one feed stream and one drain stream. Once entered into the reactor, the feeding stream is transformed into a drain stream involving different chemical composition due to the effect of concentrating solar energy. Besides, the energy balance is formulated using the total thermodynamic enthalpies of all streams including their possible loadings.^37^ The working fluids used in the simulation model of SRM could be seen in Table 1.

**Table 1:** The working fluids used in the simulation model of steam reforming of methane

Name of working fluids	Elements
Water/steam	H_2_O_(liq)_/H_2_O_(gas)_
Gas substance	CH_4_, CO, CO_2_, H_2_, H_2_O, H_2_S, N_2_, O_2_, SO_2_

IPSEpro software is used to perform the numerical investigation of the SRM process. The simulation is carried out by considering different operating parameters including temperature, pressure, chemical composition, and mass flow rates of the inlet streams. Moreover, the heat input necessary to conduct steam reforming process including side reactions such as water–gas shift reaction and the conversion of hydrocarbons is considered in this study. Besides, important factors such as pressure drop, temperature decrease and heat loss of reactor that can significantly affect the system efficiency have been investigated to improve the thermochemical reaction performance. The chemical compositions, operating parameters, and material and energy balances of all outlet material streams are calculated based on the mentioned above assumptions. Chemical equilibrium in a chemical reforming reactor could be calculated in the equilibrium constant. If the composition and mass flow rate of feed gas are specified, the composition and mass flow rate of drain gas are calculated by prescription of conversion rates or setting the concentrations in the drain.^36,37^ The conversion rate of a component *i* in dynamic equilibrium can be calculated as follows.4
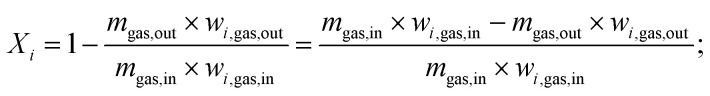
where *X*_*i*_ is a conversion rate of component *i*, *m*_gas,out_ and *m*_gas,in_ are the mass flow of outlet and inlet gases, while *w*_*i*,gas,out_ and *w*_*i*,gas,in_ are the mass fraction of outlet and inlet gases. The conversion rate is not calculated if *w*_*i*,gas,in_ = 0. The model attributes from 0 to *X*_*i*_ as an ideal value. The conversion rate of hydrocarbon species C_*x*_H_*y*_ (C_2_H_4_, C_2_H_6_, C_3_H_8_, and CH_4_) and conversion rate of steam are typically formulated in the case of steam reforming and WGS reactions.

The chemical equilibrium formulation is introduced in this section.^37^ In the chemical reactor model, the minimization of Gibbs free enthalpy as the necessary condition for the progress of chemical reactions is not considered. In general, the chemical reaction can be calculated by the following formulae.5
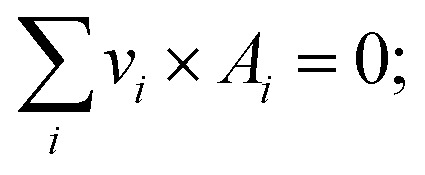
where *A*_*i*_ are all species taking part in the reaction and *v*_*i*_ are their stoichiometric coefficients.

The equilibrium constant for the partial pressures is calculated by the following formulae.6
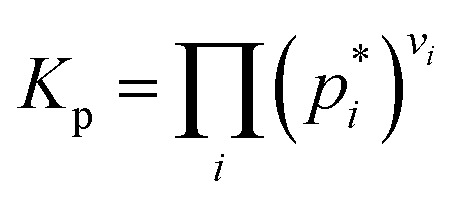


In the case of ideal gases, the equilibrium constant is only a function of temperature, which is calculated by the reaction of Gibbs free enthalpy minimization.7
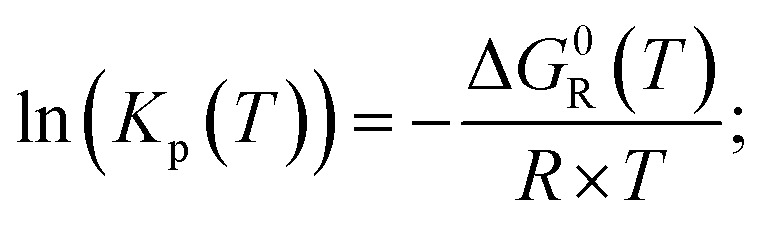
where Δ*G*^0^_R_ is the Gibbs free enthalpy at standard pressure, as calculated from the thermodynamic enthalpies and entropies of the participating species:8
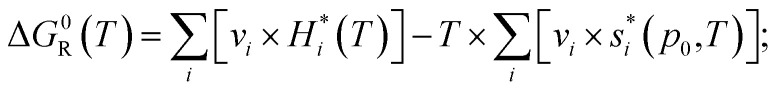


The actual partial pressure product is related to the equilibrium constant and the logarithm of the ratio, which is expressed as a model parameter:9
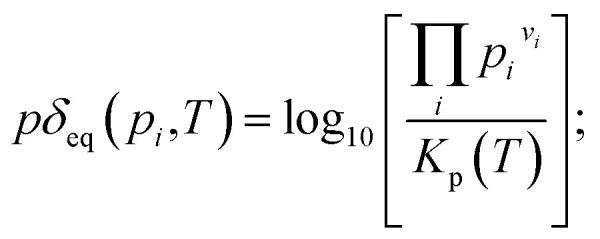
If *pδ*_eq_ < 0, then the actual state of the drain gas is still on the side of the reactants, *i.e.* further reaction is thermodynamically in direction of the products possible. If *pδ*_eq_ > 0, the actual state of the drain gas is on the side of the products. In this case, the reaction can only proceed towards the reactants, *i.e.* from right to left. If *pδ*_eq_ = 0 is prescribed, equilibrium must be fulfilled by the gas composition. In general, the equilibrium calculation requires *p*_*i*_ > 0 for in all gas species.

The operation performance of the SRM reactor is characterized by methane conversion rate, steam conversion rate, hydrogen yield, thermal efficiency of reactor and energetic upgrade factor.^38^ Methane conversion rate, steam conversion rate and hydrogen yield can be defined as follows.

Methane conversion rate:10
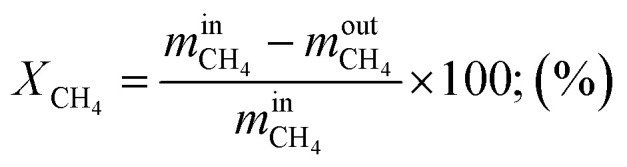


Steam conversion rate:11
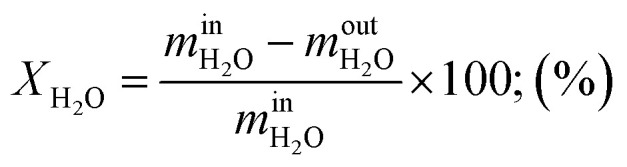


Hydrogen yield:12
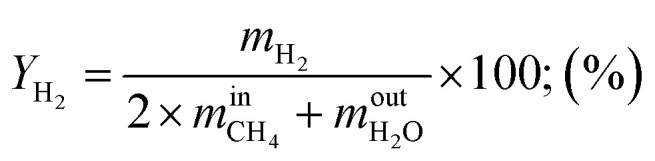
where 
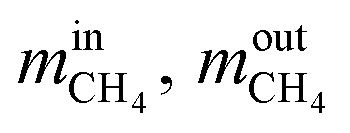
 are the mass flow of methane at the inlet and outlet of the reformer; 
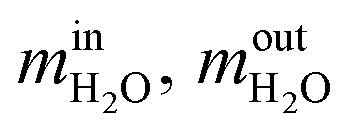
 are the mass flow of steam at the inlet and outlet of the reformer; *m*_H_2__ is mass flow of hydrogen.

The thermal efficiency of the SRM reactor can be determined by the following equation.13
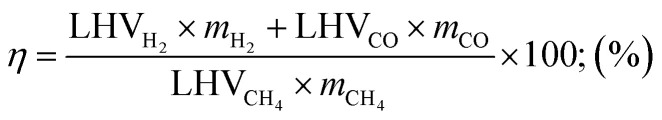
where LHV_H_2__, LHV_CO_ and LHV_CH_4__ are the lower heating value of hydrogen, carbon monoxide and methane, respectively while *m*_H_2__, *m*_CO_ and *m*_CH_4__ are the mass flow rate of hydrogen, carbon monoxide and methane, respectively.

The conversion efficiency of solar energy into chemical energy by the solar SMR is characterized by the energetic upgrade factor (*U*).^39^ The energetic upgrade factor can be determined as the ratio of the LHV of reformate gas produced to that of the feedstock processed.14
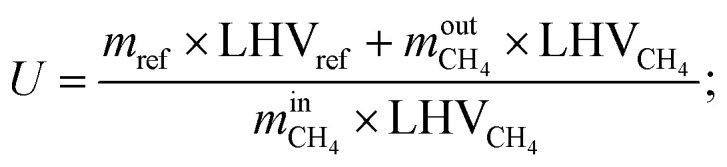
where *m*_ref_ is the mass flow rate of reformate gas; LHV_ref_ is the lower heating value of reformate gas.

### 2.2. Model of a solar field with a central receiver system

**Fig. 3:**
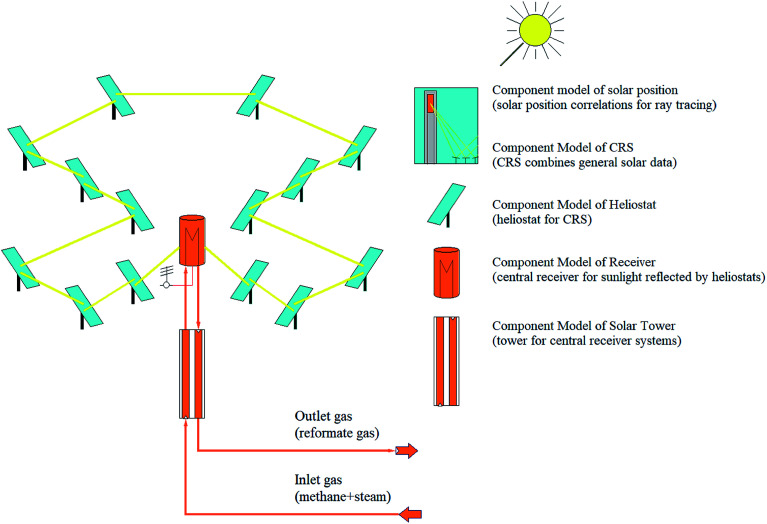
Process flow diagram of a central receiver system for solar SRM process.

SMR process is an endothermic process and requires a heat source with high potential. The solar SRM process is similar to that of conventional except for the energy source in which the solar SRM process is powered by high-temperature concentrated solar energy. According to the numerical and experimental studies, the dish and centralized tower type solar concentrating systems are convenient for solar SRM process.^40,41^ Previously, a few studies on the thermochemical hydrogen production using a CSP plant with a central receiver system have been reported in the literature.^42,43^ CSP library of IPSEpro software^44^ was used to perform the simulation model of the solar field (SF) with a central receiver system of SRM reactor concerning the data reported in the literature. The process flow diagram of the solar field with a central receiver system for solar SRM process and component models used in the simulation model are basically described in Fig. 3. The solar field with a central receiver system consists of a receiver unit, solar tower, and heliostat. Heliostat field has 14 subfields, which are located on three annular rings with their respective center given coordinates. Direct normal irradiation (DNI) is the most important parameter for performance assessment of a solar field with a centralized tower, which is prescribed as the input value for any process model of concentrated solar energy utilization.

#### 2.2.1 Heliostat

Heliostat solar field consists of a number of individual heliostats, which surround the solar tower and reflect sunlight to the central receiver system set on the top of a solar tower. Solar thermal power reflected towards a central receiver system (*q*_refl_) can be determined by the following equation.

15 *q*_refl_ = *q*_inc_ × *η*_hel_;

where *q*_inc_ is solar power incident on the total area of heliostat mirror; *η*_hel_ is the heliostat efficiency, which is composed of reflectivity and spilling.

Solar power incident on the total area of a mirror (*q*_inc_) can be determined by the following equation.

16 *q*_inc_ = DII × *A*_hel_;

where DII is direct incident irradiance; *A*_hel_ is aperture area of the heliostat.

Direct incident irradiance (DII) can be determined by the following equation.

17 DII = DNI cos *θ*;

where DNI is direct normal irradiance; *θ* is incident angle.

#### 2.2.2 Central receiver system

Central receiver at the top of the solar tower is used to transfer the sunlight reflected by heliostats to the HTF. Received summary of reflected solar power from heliostats (*q*_rec_) can be calculated as:

18 *q*_rec_ = *q*_refl_ − *q*_rec.loss_;

where *q*_rec.loss_ is the solar thermal power loss in the receiver, which is composed of the power loss reflected from receiver, radiation and convection losses of the receiver.

Solar thermal power loss in the receiver (heat loss from receiver aperture area to ambient air) can be calculated as:

19 *q*_rec.loss_ = *q*_ref.loss_ + *q*_rad.loss_ + *q*_conv.loss_;

where *q*_refl.loss_ is the power loss reflected from the receiver; *q*_rad.loss_ is the radiation loss of receiver; *q*_con.loss_ is the convection losses of the receiver. *q*_refl.loss_, *q*_rad.loss_ and *q*_con.loss_ can be obtained by ref. 45.

20 *q*_refl.loss_ = (1 − *δ*) × *q*_refl_;

21 *q*_rad.loss_ = ∑*εσ*_0_*A*_r_(*T*_wall,*i*_^4^ − *T*_amb_^4^);

22 *q*_conv.loss_ = ∑*f*_mix,*i*_*A*_r_(*T*_wall,*i*_ − *T*_amb_);

where *δ* is concerning solar absorptance of the tube panels, (0.95); *ε* is the hemispherical emittance, (0.88); *σ*_0_ is the Stefan–Boltzmann constant; *A*_r_ is the lateral surface of the tube; *f*_mix,*i*_ is the mixed convection coefficient; *T*_wall,*i*_ is the wall temperature; *T*_amb_ is the ambient air temperature.

Thermal efficiency of receiver system can be calculated as:23
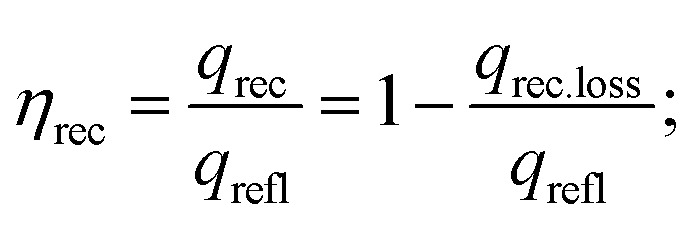


Sunlight reflected by mirrors of heliostat is focused onto the central receiver system and the temperature of HTF increases by transferring heat (absorbing energy) from the receiver. Transferred heat (*q*_trans_) to HTF can be calculated as:

24 *q*_trans_ = *m*_HTF_ × (*h*^out^_HTF_ − *h*^in^_HTF_);

where *m*_HTF_ is the mass flow rate of heat transfer fluid; *h*^out^_HTF_ and *h*^in^_HTF_ are specific enthalpy of HTF at outlet and inlet of receiver.

The receiver model is validated with experimental data from the Solar Two power plant. The actual efficiency of the receiver system is around 85.2%, which agrees well with the experimental data of Solar Two demonstrated as 86–88%.^46^ Moreover, other studies reported the efficiency of the receiver as 83–90% by Jianfeng *et al.*^47^ and 78–88% by Lata *et al.*^48^ Therefore, the precision and simulation result of the receiver system could satisfy the project modeling requirements.

**Fig. 4:**
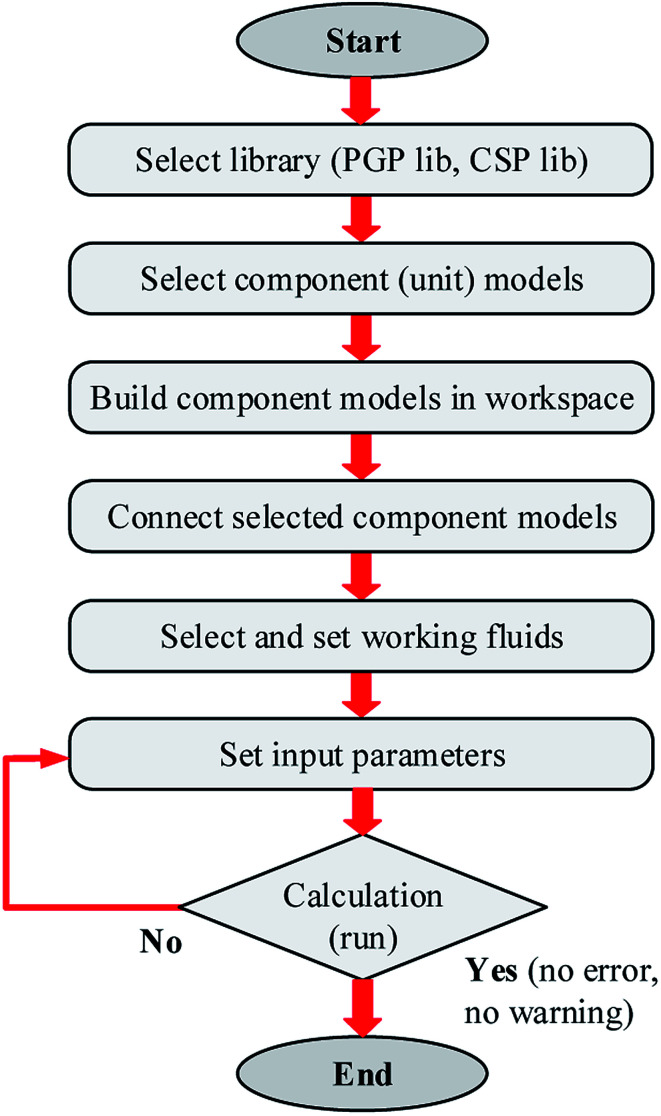
Simplified flowchart for calculating the simulation model of the SRM process.

The simplified flowchart for calculating the simulation model of solar SRM using IPSEpro software can be seen in Fig. 4. The simulation models contain a mass balance, energy balances and specific equations describing chemical conversion rates, splitting conditions, basic functional and empiric correlations.^49^ However, it is first necessary to build the component models of the SRM plant for analyzing the process flow diagram of SRM. In general, the process modeling is based on the component models provided by IPSEpro which describes the basic physical and chemical processes, builds the total SRM plant according to the sequence of components including the component models, subsystems, and complete systems.^50^

#### 2.2.3 Model validation

**Fig. 5:**
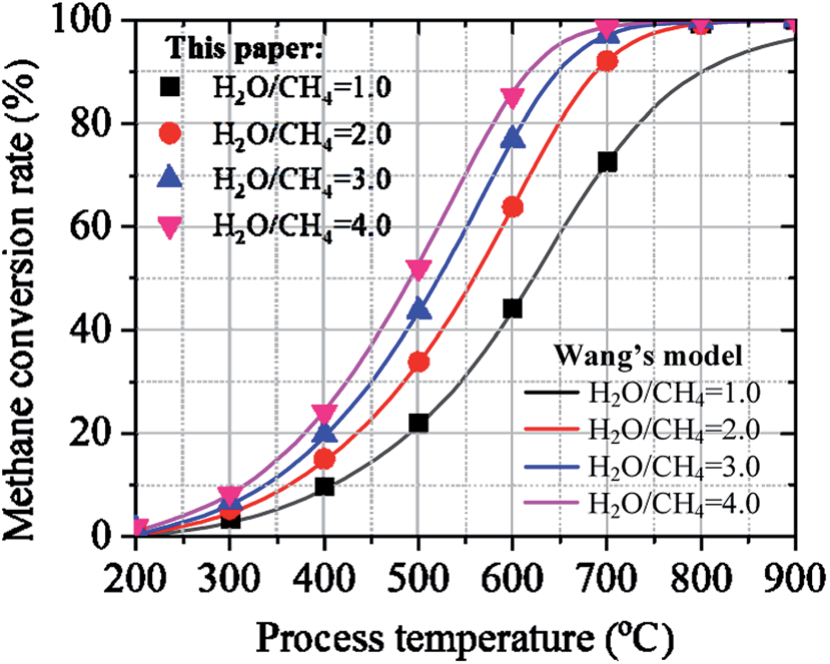
Effect of process temperature on the methane conversion rate between the results calculated in Wang's model and in this paper.

The accuracy of IPSEpro models and the validation of our simulation results were carried out and discussed under the same simulation conditions used by Wang *et al.*^51^ Wang *et al.* have investigated the effect of process temperature on the methane conversion rate under the operation conditions of 200–900 °C of process temperature, 1.02 bar of operating pressure, and 1.0–4.0 of mass flow ratio of feed gas. Fig. 5 clearly describes the comparison for the effect of process temperature on the methane conversion rate under various ratios of feed gas between the results calculated in Wang's model and in this paper. As can be seen from Fig. 5, the calculated results agreed well with those reported in Wang's model. Increasing the mass ratio of feed gas leads to an increase in the methane conversion rate at the given reaction temperature. Moreover, the methane conversion rate is increased by growing process temperature under the different mass ratios of feed gas. The simulation results of IPSEpro model from this study are accurate compared to the previous study. Moreover, the simulation results have similar behavior and pattern under the same operating conditions. According to the results, IPSEpro model can be considered for the simulation model of SRM since it satisfies the project modeling requirements.

## 3. Results and discussion

### 3.1. Material balance analysis of feed and reformate gas

According to the literature, the process modeling of the SRM can be simulated by different software with diverse mathematical models. Steady-state simulation of H_2_ production from SRM process is performed using IPSEpro process simulation software. Important factors including mass flow ratio of H_2_O/CH_4_ in the feed gas, reaction temperature, and operating pressure of the feed gas that can influence the compositions of reformate gas in the reformer are considered during the numerical analysis. A pressure range of 1.02–30 bar, temperature range of 200–1000 °C, and mass ratio of steam-to-methane (H_2_O/CH_4_) of 1.0–4.0 are the limited conditions calculating the composition of reformate gas (CH_4_, CO, CO_2_, H_2_ and H_2_O), methane conversion rate, steam conversion rate, volume flow of reformate gas, and volume flow of hydrogen gas. The material balance of feed gas into the reforming reactor can be seen in Table 2. In this study, the feeding gas is essentially a composite of CH_4_ and H_2_O. The numerical calculation is carried out by varying the ratio of H_2_O/CH_4_ from 1.0 to 4.0 while maintaining constant the process temperature at 800 °C and the pressure at 1.02 bar.

**Table 2:** The material balance of feed gas into the reforming reactor under 800 °C at 1.02 bar

Item	Methane (CH_4_)		Steam (H_2_O)	
Unit	kg h^−1^	N m^3^ h^−1^	kg h^−1^	N m^3^ h^−1^
Ratio of H_2_O/CH_4_ = 1.0	1.00	1.40	1.00	1.24
Ratio of H_2_O/CH_4_ = 1.5	1.00	1.40	1.50	1.86
Ratio of H_2_O/CH_4_ = 2.0	1.00	1.40	2.00	2.49
Ratio of H_2_O/CH_4_ = 2.5	1.00	1.40	2.50	3.11
Ratio of H_2_O/CH_4_ = 3.0	1.00	1.40	3.00	3.73
Ratio of H_2_O/CH_4_ = 4.0	1.00	1.40	4.00	4.98

Moreover, the input value of the concentration of CH_4_ and H_2_O is clearly described in Table 3 by considering different mass ratios of H_2_O/CH_4_. The volume flow rate of reformate gas at normal condition changes with the variation in the operating conditions. Increasing H_2_O/CH_4_ ratio from the feed gas and the reacting medium resulted in an increase in the volume flow rate of reformate gas at normal conditions. Thus, the concentration of H_2_O is responsible for the density of reformate gas in the reactor. As for H_2_ yield, both mass flow rate and volume flow rate are affected by the change in the operating conditions at normal conditions. It was found that the increase in the ratio of H_2_O/CH_4_ leads to a slight increase in hydrogen production at the given reaction temperature. By considering the operating temperature at 800 °C, an important amount of hydrogen gas is observed at a 4.0 ratio of H_2_O/CH_4_ and 1.02 bar of operating pressure. The ratio of H_2_O/CH_4_ = 1.0 corresponding to 1.00 kg of methane and 1.00 kg of steam produces about 0.32 kg of hydrogen gas while 0.43 kg is obtained for the same mass of mixture gas considering the ratio of H_2_O/CH_4_ = 4.0. Every 2 kg of methane produces about 1 kg of hydrogen gas under the temperature range of 327–827 °C, at a pressure of 1.02 bar when the ratio of H_2_O/CH_4_ = 4.5.^34^ As can be seen in Table 3, the mass ratio of feed gas and the WGS reaction is slightly influenced by hydrogen production. H_2_O and CH_4_ are the most abundant source of H_2_. Besides, the WGS reaction results in higher H_2_ production at certain operating conditions. Combining these processes could evidently result in higher H_2_ production with a slightly increasing mass ratio of H_2_O/CH_4_. Therefore, the ratio of H_2_O/CH_4_, operating temperature are important key parameters that should be carefully investigated to optimize the process of hydrogen production *via* SRM.

**Table 3:** The material balance of reformate gas from the reforming reactor at 1.02 bar

Item	Reformate gas		Hydrogen gas	
Unit	kg h^−1^	N m^3^ h^−1^	kg h^−1^	N m^3^ h^−1^
Ratio of H_2_O/CH_4_ = 1.0	2.00	4.98	0.32	3.53
Ratio of H_2_O/CH_4_ = 1.5	2.50	5.98	0.37	4.09
Ratio of H_2_O/CH_4_ = 2.0	3.00	6.65	0.39	4.38
Ratio of H_2_O/CH_4_ = 2.5	3.50	7.29	0.41	4.51
Ratio of H_2_O/CH_4_ = 3.0	4.00	7.91	0.42	4.61
Ratio of H_2_O/CH_4_ = 4.0	5.00	9.16	0.43	4.75

### 3.2. Parameters study

#### 3.2.1 Effect of mass flow ratio on feed gas

**Fig. 6:**
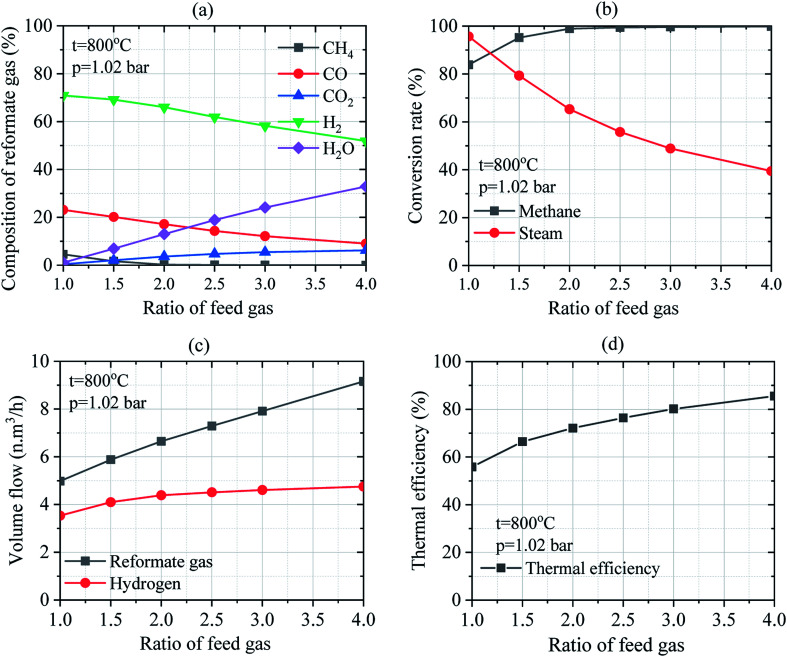
Effect of the mass flow ratio of feed gas on the SRM process: (a) composition of reformate gas; (b) conversion rates of methane and steam; (c) the volume flow of reformate gas and hydrogen; (d) thermal efficiency of reforming reactor.

The effect of the mass flow ratio of feed gas (steam-to-methane) on the SRM process was investigated under the temperature of 800 °C and 1.02 bar of operating pressure. Composition of reformate gas is considered in wet and dry mass. After the reactor, the composition of reformate gas contains water vapor, which is called wet mass. The mass of reformate gas after the purification process is called the dry mass. The quality of the reformate gas is significantly improved after the purification process. Fig. 6(a) indicates the effect of the mass ratio of H_2_O/CH_4_ on the composition of reformate gas from the reformer in wet mass. By considering the wet mass, the concentration of CH_4_, CO, and H_2_ in the reformate gas decrease while the concentration of CO_2_ and H_2_O increase as the ratio of H_2_O/CH_4_ increases. Analysis of the composition of reformate gas results in the decrease in CH_4_ concentration from 4.523–0.023%, CO from 23.126–9.028%, H_2_ from 70.897–51.866%, and the increase in CO_2_ from 0.379–6.196% and H_2_O from 1.075–32.888% when the ratios of H_2_O/CH_4_ was increased from 1.0–4.0. Besides, it was observed that the decrease in the concentration of CH_4_ form 4.572–0.034%, CO from 23.377–13.452%, and the increase in CO_2_ and H_2_ concentration from 0.383–9.232% and 71.667–77.283%, respectively when the ratios of H_2_O/CH_4_ was increased from 1.0–4.0 in dry mass. Thus, CH_4_ and CO concentrations in the reformate gas decrease while CO_2_ and H_2_ concentrations increase as the ratio of H_2_O/CH_4_ increases by considering dry mass. The increase in the concentration of CO_2_ along with that of H_2_ could be attributed to the reverse water–gas shift reaction leading to significant H_2_O and CO conversion into CO_2_ and H_2_.

Regarding the effect of the ratio of H_2_O/CH_4_ on the conversion rate of methane and steam as depicted in Fig. 6(b), methane conversion rate increases compared to steam conversion rate which decreases when the ratio of H_2_O/CH_4_ was increased at a given temperature and pressure. The increase in the methane conversion rate from 83.86% up to 99.85% and the decrease in steam conversion rate from 95.69% to 39.44% is obtained at 800 °C and 1.02 bar of operating temperature and pressure, respectively. Consequently, higher volume flows of reformate gas and hydrogen was obtained at higher ratios of steam-to-methane as shown in Fig. 6(c). By considering the operating temperature of 800 °C and pressure of 1.02 bar, the volume flow of reformate gas and that of hydrogen increase to 4.98–9.16 N m^3^ h^−1^ and 3.53–4.75 N m^3^ h^−1^, respectively. Thus, reformate gas, as well as hydrogen gas were thermodynamically favored by the higher ratio of H_2_O/CH_4_. It could be observed that the mass ratio of feed gas has a big effect on steam reforming and WGS process. Besides, the effect of the ratio of H_2_O/CH_4_ on the thermal efficiency of reforming reactor was investigated under the operating conditions of 800 °C of the reaction temperature and at 1.02 bar of pressure as shown in Fig. 6(d). It was found that the thermal efficiency of the reforming reactor increases from 55.79% to 85.64% as the ratio of H_2_O/CH_4_ increases as well. It can be reported that the thermal efficiency of the reforming reactor is thermodynamically favored at a higher ratio of steam-to-methane.

#### 3.2.2 Effect of process temperature

**Fig. 7:**
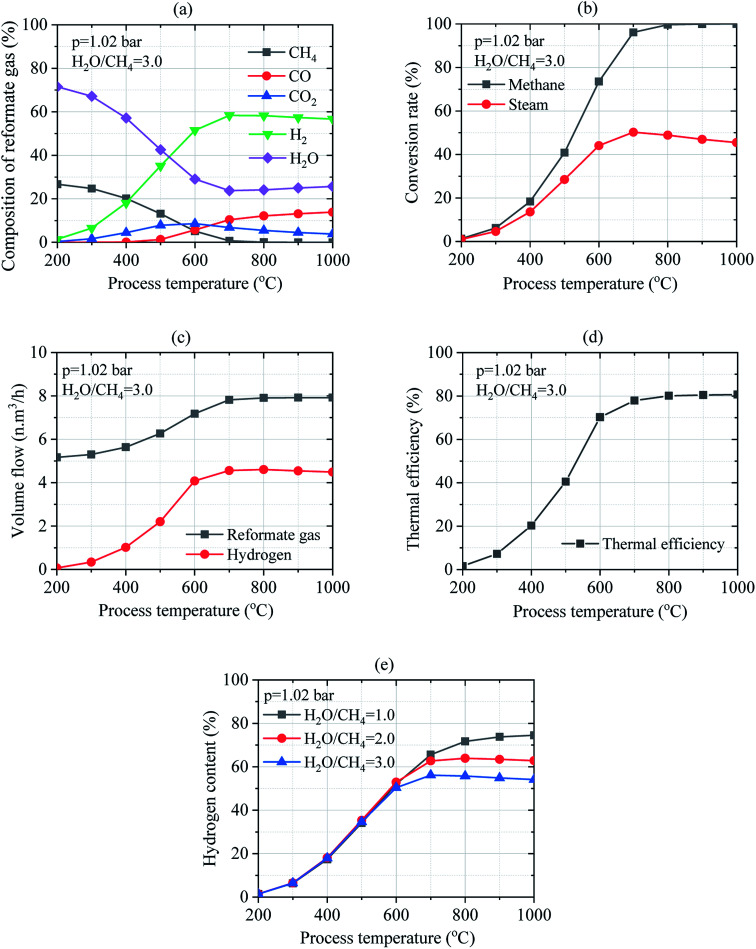
Effect of temperature on the SRM process: (a) composition of reformate gas; (b) conversion rates of methane and steam; (c) the volume flow of reformate gas and hydrogen gas; (d) thermal efficiency of reforming reactor; (e) hydrogen content in the reformate gas under the different mass ratios of feed gas.

The effect of temperature on the SRM process was investigated for the pressure of 1.02 bar and temperature ranging from 200 °C to 1000 °C under the ratio of steam-to-methane of 3.0. In theoretical, two moles of steam can fully transform one mole of methane. However, the SMR process is usually carried out under an excess ratio of H_2_O/CH_4_ (more than 3.0) in practice. Considering the reformer in wet mass as indicated in Fig. 7(a), the concentration of H_2_ and CO_2_, in the reformate gas increases while the contents of CH_4_, H_2_O and CO decrease with the increase in the process temperature at H_2_O/CH_4_ = 3.0. However, when process temperature increases, H_2_ content first increases, reaching a maximum value, and then slowly decreases. Higher conversion of CH_4_ and H_2_O leading to the variation of CH_4_ and water vapor concentration to 26.684–0.0% and 71.528–25.685%, respectively increases the yield of 1.430–56.683% of H_2_, 0.358–3.788% of CO_2_, and 0.0–13.844% of CO in the reformate gas. In the contrast to wet mass, dry mass results in a great amount of reformate gas with H_2_ concentration of 5.022–76.274%, CO_2_ of 1.257–5.097%, and CO of 0.0–18.629% at the ratio of H_2_O/CH_4_ = 3.0 with methane concentration varied to 93.72–0.0%.

Thus, the process temperature is an important key factor that significantly influences the conversion rates of methane and steam. As shown in Fig. 7(b), methane and steam conversion rates could be thermodynamically favored between 200–1000 °C since higher conversion rates of methane and steam reported to 1.32–100.0% and 0.99–45.47%, respectively were obtained at H_2_O/CH_4_ = 3.0. Moreover, the volume flow of reformate gas and hydrogen gas reported to 5.17–7.92 N m^3^ h^−1^ and 0.074–4.492 N m^3^ h^−1^, respectively was observed in Fig. 7(c) when the process temperature was increased. Therefore, increasing the process temperature results in the increase in the thermal efficiency of the reforming reactor from 1.58% up to 80.68% as depicted in Fig. 7(d). Operating temperature ranging in 700–800 °C could thermodynamically favor the SMR process at H_2_O/CH_4_ = 3.0. Results show that the process temperature has a big effect on steam reforming and WGS process. Fig. 7(e) shows the hydrogen content in reformate gas under the various mass ratio of feed gas and process temperature. The hydrogen content can increase by reducing the mass ratio of feed gas. It's depending on the mass flow of reformate gas. Moreover, the hydrogen content is increased by increasing process temperature under the different ratios of feed gas. Besides, it is pertinent to mention that the volume flow of hydrogen is increased when the mass ratio of the feed gas is increased.

#### 3.2.3 Effect of process pressure

**Fig. 8:**
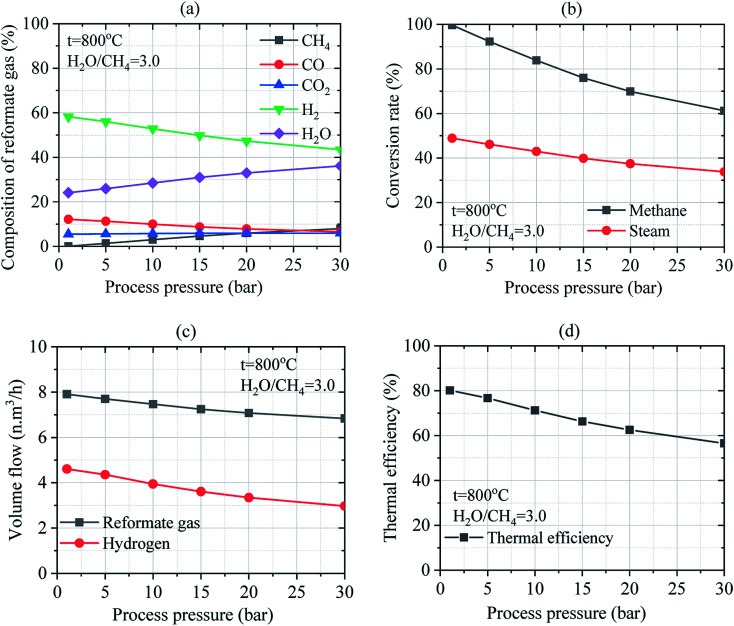
Effect of pressure on the SRM process: (a) composition of reformate gas; (b) conversion rates of methane and steam; (c) the volume flow of reformate gas and hydrogen gas; (d) thermal efficiency of reforming reactor.

The effect of process pressure on the SRM process was investigated for the temperatures of 800 °C and operating pressure ranging from 1.02 bar to 30 bar under a mass flow ratio of steam-to-methane of 3.0. By considering the ratio of H_2_O/CH_4_ = 3.0, the composition of the reformate gas includes 0.059–7.921% of CH_4_, 12.148–6.580% of CO, 5.446–5.925% of CO_2_, 58.228–43.437% of H_2_, and 24.119–36.137% of water vapor at wet mass as shown in Fig. 8(a). However, the change in the composition of the reformate gases of 0.078–12.403% of CH_4_, 16.009–10.303% of CO, 7.177–9.278% of CO_2_, and 76.736–68.016% of H_2_ was obtained at the same ratio of H_2_O/CH_4_ = 3.0 at dry mass. Regarding the composition of the reformate gases, increasing the process pressure significantly affects the global yield of reformate gases. The concentrations of CH_4_, CO_2_ and H_2_O increase while those of CO and H_2_ decrease in reformate gases when the process pressure was increased by considering the wet mass at the ratio of H_2_O/CH_4_ = 3.0. Besides, the conversion rate of methane and steam decrease as the process pressure increases as shown in Fig. 8(b). At H_2_O/CH_4_ = 3.0, the conversion rates of methane and steam decreased from 99.67–61.22% and 48.86–33.77%, respectively when the operating pressure was increased from 1.02–30 bar. Thus, CH_4_ and steam conversion rates are thermodynamically favored at low operating pressure. At the same time, as can be seen in Fig. 8(c), the volume flow of reformate gas and hydrogen gas gradually decrease from 7.91–6.84 N m^3^ h^−1^ and 4.61–2.97 N m^3^ h^−1^, respectively due to the decrease in the thermal efficiency of reforming reactor associated with the increase in the process pressure. Moreover, the thermal efficiency of the reforming reactor could slightly decrease from 80.13–56.54% with the increase in the operating pressure as depicted in Fig. 8(d). Pressure has a low effect on steam reforming and WGS process. However, the process pressure has operational advantages.

### 3.3. Assessment of solar heat input for steam reforming methane process

The assessment of solar heat input on the SRM process was investigated *via* a concentrated solar power library (CSP lib) of IPSEpro software. The numerical calculation is carried out by varying the temperature from 600 °C to 900 °C while maintaining constant the ratio of H_2_O/CH_4_ at 4.0 and the pressure at 1.02 bar. The combined steam methane reforming and water–gas shift stoichiometric reaction for a 4 : 1 steam to methane ratio at 800 °C is given by the mass fraction (kg kg^−1^) in eqn (25).25
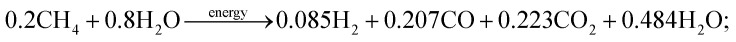


The input values of material balance of feed gas, estimated values of material balance of reformate gas, the composition of reformate gas, and operation performance of reforming reactor are clearly described in Table 4 at 800 °C. The SRM process is carried out at 30.1 MW of heat input for processing 5.0 tons of methane and 20.0 tons of steam under 800 °C of temperature and 1.02 bar of pressure. The results show that 2.14 tons of hydrogen can be produced from 5.0 tons of methane using 30.1 MW of solar power can be achieved.

**Table 4:** Material balance of feed and reformate gas under 800 °C at 1.02 bar

Item	Unit		Value
Material balance of feed and reformate gases	The mass flow rate of methane	tons per h	5.00
	The mass flow rate of steam	tons per h	20.00
	The ratio of H_2_O/CH_4_	—	4.00
Composition of reformate gas	Methane (CH_4_)	%	0.023
	Steam (H_2_O)	%	32.888
	Carbon monoxide (CO)	%	9.028
	Carbon dioxide (CO_2_)	%	6.196
	Hydrogen (H_2_)	%	51.866
Operation performance of SRM reactor	Methane conversion rate	%	99.85
	Steam conversion rate	%	39.44
	Hydrogen yield	%	61.16
	The thermal efficiency of a chemical reactor	%	85.75
	Energetic upgrade factor	—	1.236
	Required solar heat input for SRM process	kW	30 100

**Fig. 9:**
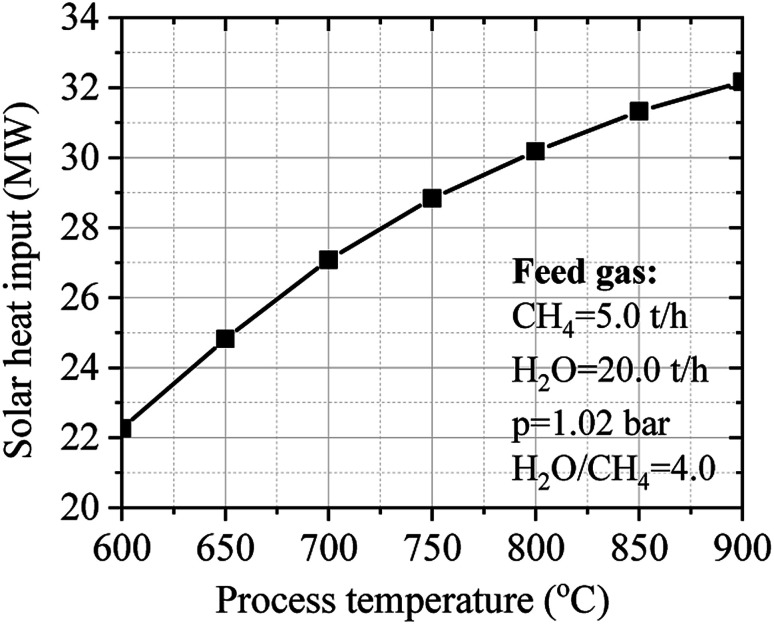
Effect of temperature on the solar heat input of the SRM process.

Fig. 9 shows the influence of temperature on the solar heat input of the SRM process. The process temperature is directly dependent on the solar heat input of the SRM process. Increasing the process temperature until to desired thermochemical conversion temperature leading to good operating performance can be obtained by increasing the solar heat input of the SRM process. However, the process temperature should be limited to 900 °C to ensure the long-term durability and stability of catalysts. Noted that in practice, most cases of steam reforming reactors operate around 800 °C.

**Fig. 10:**
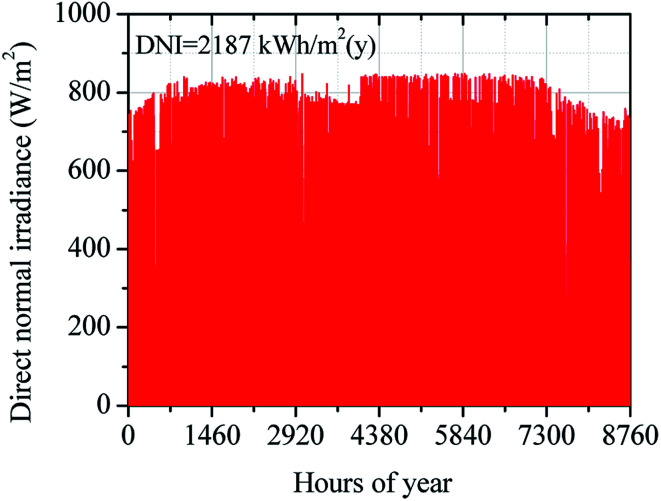
Annual DNI distribution in Dalanzadgad city.

In this study, the climate data of Dalanzadgad city in Omnogobi province (43.35° S, 104.41° E) was offered by NREL's standard system advisor model library (SAM). To create the results closer to real operation conditions, the simulation model of the solar SRM system is performed based on climate data in a typical year of Dalanzadgad city given in Table 5.^50^ Variation of annual DNI in a typical year of Dalanzadgad city is shown in Fig. 10. The annual DNI distribution of Dalanzadgad city was around 2187 kW h m^2^. The design-point value of DNI is taken as 0.60 kW m^−2^ based on climate data of Dalanzadgad city in 2017. The main parameters and operation performance of the solar field were estimated based on this design-point value of DNI.

**Table 5:** Climate data of Dalanzadgad city

Item	Unit	Value
Annual total of DNI	kW h m^−2^	2187.5
Daily DNI	kW h m^−2^	5.99
Design value of DNI	kW m^−2^	0.60
Elevation	m	1460
Average temperature	°C	6.2
Average wind speed	m s^−1^	3.5
Average relative humidity	%	40

**Fig. 11:**
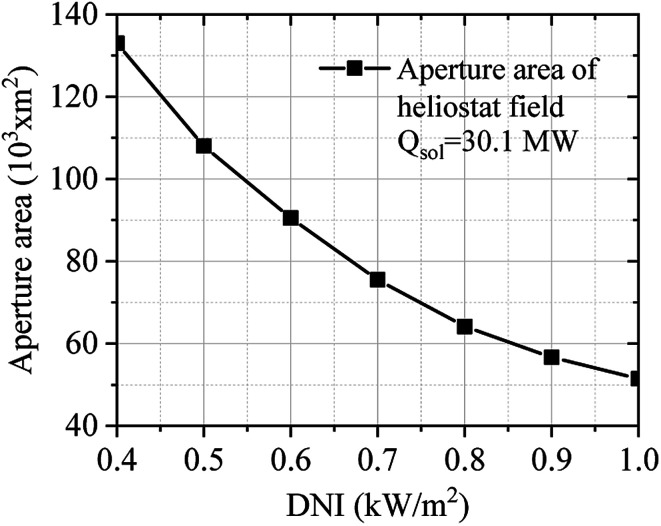
Effect of DNI on the aperture area of the solar field.

Fig. 11 describes the effect of DNI on the aperture area of the solar field. The aperture area of the solar field was calculated considering the required solar heat (30.1 MW) of the SRM process. DNI is an important key factor that significantly influences the aperture area of the solar field. The DNI condition was varied between 0.40 to 1.00 kW m^−2^. As shown in Fig. 11, the aperture area of the solar field decreases when DNI increases.

The main parameters and operating performance of the solar field with a central receiver system are summarized in Table 6. Numerical simulation of the solar field was performed in the off-design regime (variation of DNI). As can be seen in Table 6, the DNI is a key parameter affecting the operation performance of the solar field with a central receiver system.

**Table 6:** The main parameters and operation performance of the solar field

Item	Unit	Value							
DNI	kW m^−2^	1.00	0.80	0.70	0.60	0.50	0.40	0.30	0.20
DII	kW m^−2^	0.780	0.564	0.546	0.423	0.352	0.282	0.234	0.146
Solar tower height	m	50	50	50	50	50	50	50	50
Receiver aperture area	m^2^	30	30	30	30	30	30	30	30
Aperture area of single heliostat	m^2^	60	60	60	60	60	60	60	60
Number of heliostat	—	1550	1550	1550	1550	1550	1550	1550	1550
Effective aperture area of solar field	m^2^	93 000	93 000	93 000	93 000	93 000	93 000	93 000	93 000
Efficiency of receiver	%	85.18	82.66	80.89	78.57	75.34	68.95	60.97	52.50
Thermal efficiency of solar field	%	58.8	57.1	55.9	54.5	52.1	47.6	42.0	36.2
Transferred heat to SRM process	MW	54.69	42.31	36.18	30.10	24.35	17.45	11.57	6.74

**Fig. 12:**
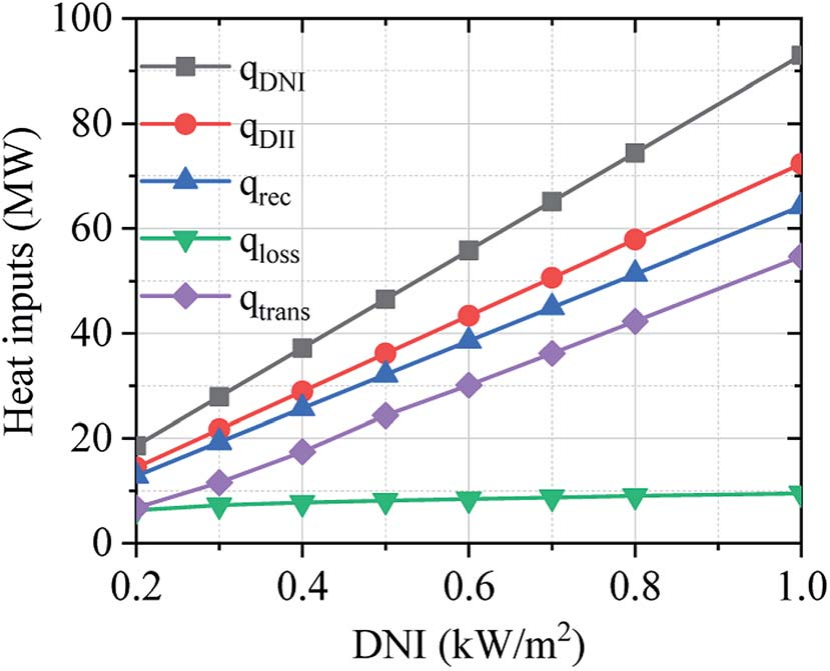
Effect of DNI on the operation performance of solar field: *q*_DNI_ – DNI on effective aperture area; *q*_DII_ – DII hitting the mirror on heliostat; *q*_rec_ –reflected solar radiation from heliostat; *q*_trans_ –transferred heat to SRM process; *q*_loss_ –heat loss of receiver.

Fig. 12 shows the operation performance of the solar field in the off-design regime. The operation performance of the solar field has been calculated considering the design-point aperture area (*A*_SF_ = 93 000 m^2^), and the DNI changes from 0.20 to 1.00 kW m^−2^. Operation performance of the solar field mainly depends on DNI value. The solar field has good performance under higher DNI condition, but the optical and thermal losses are increased.

**Fig. 13:**
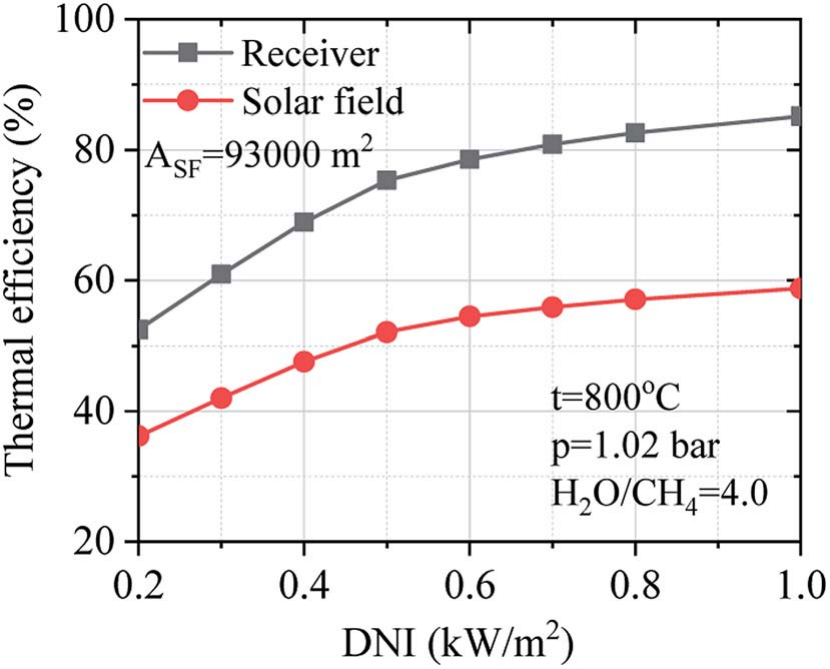
Effect of DNI on the thermal efficiency of the receiver system and solar field.

Fig. 13 describes the effect of DNI on the thermal efficiency of the receiver and solar field under the variation of DNI. The thermal efficiency of the receiver and solar field have been obtained considering the design-point aperture area, and the DNI changes from 0.20 to 1.00 kW m^−2^. The thermal efficiency of the receiver and solar field are not linear correlation on the DNI. The thermal efficiency of the receiver and solar field slightly decrease by 9.8% and 6.7% respectively from 1.00 to 0.50 kW m^−2^. Besides, the thermal efficiency of the receiver and solar field were drastically decreased between 0.40 to 0.20 kW m^−2^.

**Fig. 14:**
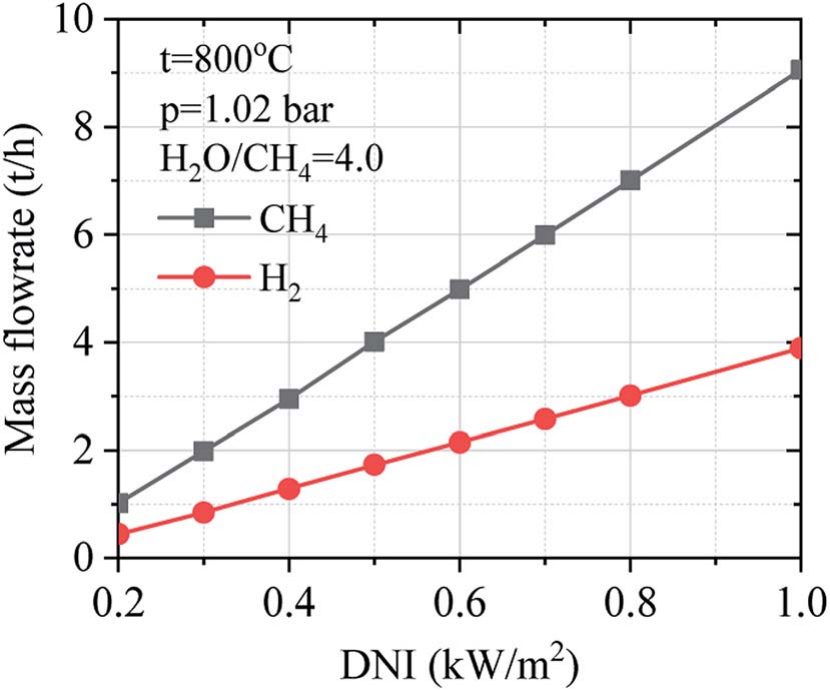
Effect of DNI on the mass flow of methane and hydrogen.

Fig. 14 describes the effect of DNI on the mass flow of methane and hydrogen gas under the variation of DNI. It could be observed that solar SRM process has higher operation performance under higher DNI conditions. The mass flow of hydrogen gas increases when the DNI value grows.

## 4. Conclusion

This paper presented the simulated results of the solar SRM process for producing low-carbon hydrogen using concentrated solar energy. The CSP technology with the centralized tower was adopted in the simulation model. The solar concentrating system with a centralized tower is the most promising CSP technology for using high temperature and large-scale application. The numerical calculation of solar SRM was considered with the process temperature and pressure varied from 200 °C to 1000 °C and 1.02 bar to 30 bar, respectively at the mass ratio of steam-to-methane ranging from 1.0 to 4.0. Favorable operating conditions of solar SRM process were high temperature and low pressure considering the higher mass ratio of feed gas. The steam methane reforming reaction and WGS reaction were considered and the effects of different operating conditions on the SRM process were investigated from the perspective of finding preliminarily optimized and validated results for solar SRM process. The following conclusions have been drawn:

(1) Considering WGS reaction in the process of steam methane reforming could result in low-carbon hydrogen formation due to the higher conversion rate of H_2_O and CO in the reformate gases;

(2) The operating conditions including temperature, pressure, and the mass ratio of H_2_O/CH_4_ have significant effects on the thermal efficiency of the SRM process and the yield of reformate gases and hydrogen;

(3) The optimum reaction temperature of the SRM process is limited to 800 °C at low operating pressure and H_2_O/CH_4_ = 3.0. Increasing the process temperature until to desired thermochemical conversion temperature leading to good operating performance can be reached by increasing the solar heat input of SRM process;

(4) Correlation between the effective aperture area of the solar field and DNI can be considered as a key parameter affecting the investment cost of the solar field with a central receiver system.

(5) The variation of DNI is a stronger impact on the operation performance of the solar SRM process.

(6) Higher operation performance of solar SRM process was obtained under higher DNI conditions.

## Abbreviations and symbols

NG: Natural gas
R&D: Research and development
SRM: Steam reforming of methane
DRM: Dry reforming of methane
POX: Partial oxidation
ATR: Auto-thermal reforming
WGS: Water–gas shift
PSA: Pressure swing adsorption
IPSEpro: Integrated process simulation environment program
PGP lib: Pyrolysis and gasification process library
CSP lib: Concentrated solar power library
CRS: Central receiver system
DNI: Direct normal irradiation
DII: Direct incident irradiation
*X*_*i*_
: The conversion rate of component *i*
*X*_CH_4__
: Methane conversion rate, (%)

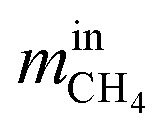

The mass flow rate of methane inlet reforming reactor, (kg h^−1^)

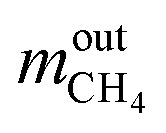

The mass flow rate of methane outlet reforming reactor, (kg h^−1^)
*X*_H_2_O_
: Steam conversion rate, (%)

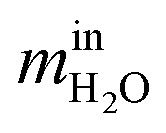

The mass flow rate of steam inlet reforming reactor, (kg h^−1^)

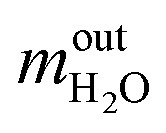

The mass flow rate of steam outlet reforming reactor, (kg h^−1^)
*Y*_H_2__
: Hydrogen yield, (%)
*m*_H_2__
: The mass flow rate of hydrogen, (kg h^−1^)
*η*
: The thermal efficiency of the reforming reactor, (%)
LHV_H_2__: Lower heating value of hydrogen, (kJ kg^−1^)
LHV_CH_4__: Lower heating value of methane, (kJ kg^−1^)
LHV_CO_: Lower heating value of carbon monoxide, (kJ kg^−1^)
*m*_CO_
: The mass flow rate of carbon monoxide, (kg h^−1^)
*m*_CH_4__
: The mass flow rate of methane, (kg h^−1^)
*U*
: Energetic upgrade factor
*q*_refl_
: Solar thermal power reflected towards a central receiver system, (kW)
*q*_inc_
: Solar thermal power incident on the total area of heliostat mirror, (kW)
*η*_hel_
: The heliostat efficiency, (%)
*A*_hel_
: Aperture area of the heliostat, (m^2^)
*θ*
: The incident angle, (°)
*q*_rec_
: Received summary of reflected solar power from heliostats, (kW)
*η*_rec_
: Thermal efficiency of receiver system, (%)
*q*_rec.loss_
: Solar thermal power loss in the receiver, (kW)
*q*_ref.loss_
: Solar power loss reflected from the receiver, (kW)
*q*_rad.loss_
: Radiation loss of receiver, (kW)
*q*_con.loss_
: Convection losses of the receiver, (kW)
*q*_trans_
: Transferred heat to heat transfer fluid, (kW)
*δ*
: Concerning solar absorptance of the tube panels
*ε*
: The hemispherical emittance
*σ*_0_
: The Stefan–Boltzmann constant, W (m^−2^ K^−4^)
*A*_r_
: The lateral surface of the tube, (m^2^)
*f*_mix,*i*_
: The mixed convection coefficient
*T*_wall,*i*_
: The wall temperature, (°C)
*T*_amb_
: The ambient air temperature, (°C)
*m*_HTF_
: The mass flow rate of heat transfer fluid, (kg h^−1^)
*h* ^out^_HTF_
: Specific enthalpy of HTF at outlet of receiver, (kJ kg^−1^)
*h* ^in^_HTF_
: Specific enthalpy of HTF at inlet of receiver, (kJ kg^−1^)
CH_4_: Methane
H_2_O: Steam
H_2_: Hydrogen
CO: Carbon monoxides
CO_2_: Carbon dioxide
H_2_O/CH_4_: Mass flow ratio of steam-to-methane

## Conflicts of interest

There are no conflicts to declare.

## Supplementary Material
